# Psilocybin-Assisted Therapy for Adolescent Anorexia Nervosa: Clinical Considerations and Emerging Models of Care

**DOI:** 10.1007/s11920-026-01679-z

**Published:** 2026-05-14

**Authors:** Jamarie A. Geller, Rachel Pacilio, Amanda E. Downey, Anya Ragnhildstveit, Marissa Raymond-Flesch, Stephanie Knatz-Peck, Natalie Gukasyan

**Affiliations:** 1 Department of Psychiatry, The University of Michigan, 1500 E Medical Center Dr, Ann Arbor, MI 48109 USA; 2 Center for Psychedelic Research, Pneuma Science, Draper, UT USA; 3 Department of Pediatrics, University of California, San Francisco, San Francisco, CA USA; 4 Department of Psychiatry and Behavioral Sciences, University of California, San Francisco, CA USA; 5 Cambridge Psychedelic Research Group, Cambridge, England, UK; 6 Department of Psychiatry, University of Cambridge, Cambridge, England, UK; 7 Philip R. Lee Institute for Health Policy Studies, University of California, San Francisco, San Francisco, CA USA; 8 Department of Psychiatry, University of California, San Diego, San Diego, CA USA; 9 New York State Psychiatric Institute, Columbia University Medical Center, New York, NY USA

**Keywords:** Anorexia nervosa, Psilocybin, Psychedelic therapy, Adolescent psychiatry

## Abstract

**Purpose of Review:**

There is growing evidence that psychedelics and associated treatment modalities may offer therapeutic benefits across a range of psychiatric conditions. Anorexia nervosa (AN), a serious and often treatment-resistant illness associated with significant morbidity and mortality, is one such condition for which psilocybin-assisted therapy (PAT) may hold promise.

**Recent Findings:**

To date, research on PAT for AN has focused primarily on adults, despite the fact that AN frequently begins in adolescence, and early age of onset portends poorer prognosis, including more severe AN, more lifetime psychiatric comorbidity, and greater life difficulties.

**Summary:**

Given these risks, an exploration of the theoretical potential of PAT for adolescent populations is warranted. Important considerations include biological implications, developmental stage, and consent. Adaptations to the current models of psilocybin-assisted therapy in studies of adults are proposed, and emerging models that address the unique challenges of these patients are discussed.

## Introduction

Growing empirical evidence supports the transdiagnostic potential of classic psychedelics, such as psilocybin-containing mushrooms and other 5-hydroxytryptamine_2A_ serotonin (5-HT2A) agonists, for treating a range of neuropsychiatric conditions [1]. These include major depressive disorder (MDD), substance use disorders (SUD), and more recently, obsessive-compulsive disorder (OCD), prompting interest in potential applications for eating disorders (EDs), namely anorexia nervosa (AN) [2–5]. Psilocybin-assisted therapy (PAT), or the adjunctive use of psilocybin with psychotherapy, has shown promise in targeting core psychopathology as well as maximizing and sustaining clinical benefits [6, 7]. While most studies investigating PAT for AN have focused on adult populations, extending this research to adolescents, a group with unique developmental, clinical, and ethical considerations, deserves careful exploration [8].

AN is a severe illness characterized by restricted energy intake, intense fear of weight gain, and body image disturbance [9]. It is the most prevalent ED among adolescents, with an estimated lifetime prevalence of 0.3–1.7% and point prevalence of 0.2–0.6% [10–12]. The onset of AN typically begins in mid-to-late adolescence, most commonly between the ages of 15 and 19 [13]. Earlier age of onset is associated with worse outcomes including longer illness duration, greater psychiatric comorbidity, and poorer quality of life [14].

Medical complications due to malnutrition in adolescents with AN are also well documented across body weights and include cardiac abnormalities (e.g., bradycardia, hypotension, and arrhythmias), endocrine disruption (e.g., arrested growth and puberty), impaired bone accretion, and even slowed brain development and loss of gray matter [15–17]. Psychiatric comorbidities are also prevalent, especially anxiety and mood disorders, which can exacerbate AN symptoms and drive chronicity [18]. Adolescents with AN often experience substantial psychosocial impairment and relapse following initial treatment is common, with approximately 20% developing chronic symptoms [19]. Moreover, mortality rates for AN remain among the highest of any psychiatric disorder. By some estimates, risk of death is up to 10%, five times higher in patients diagnosed with AN than in the general population when matched for age and sex [20–22]. Importantly, there is evidence that AN increases the risk of suicidal ideation, particularly in younger patients, and completed suicide is a leading cause of mortality in this population [23–24].

The ego-syntonic nature of the disease often results in resistance to treatment, lack of desire for recovery, and active attempts to continue disordered behaviors due to the inherent lack of insight that worsens as the disease progresses [25]. Malnutrition and its myriad cognitive consequences can further exacerbate unwillingness and/or inability to engage in meaningful treatment. On a larger scale, the lack of access to necessary interdisciplinary, evidence-based treatment is an unfortunate reality for many patients with AN, which can further hinder recovery, even for those who are motivated to engage in treatment.

Despite the increasing prevalence and severity of adolescent AN, current treatment options remain limited. Exploring innovative therapeutic approaches is, therefore, imperative. The present review discusses the theory, rationale, clinical considerations, and emerging models of PAT for adolescents with AN, while highlighting existing knowledge gaps in the field. Key recommendations for designing future psychedelic studies involving adolescents with AN, and shaping clinical guidelines and policy, are provided.

## Current Treatments

Timely weight restoration and psychosocial interventions are the mainstays of treatment for individuals with AN. In adolescents, no single psychotherapy modality has demonstrated clear superiority; however, Family-Based Therapy (FBT) is often considered the most efficacious, especially for younger patients with a shorter duration of illness, with around 40% of adolescents achieving remission [26–28]. FBT is an intensive psychotherapy intervention characterized by parent-led weight restoration with progressive return of responsibility for intake to the young person. It requires significant buy-in and consistency on the part of the caregiver/s, who are the primary agents of change. Although FBT is thought to be equally effective across demographic and clinical groups, many patients lack access both to the therapy itself and support required to engage in this treatment effectively, and it may be especially difficult for single-parent and low-resource households to implement [26, 29]. Unfortunately, some literature suggests that even gold-standard, evidence-based behavioral treatments often do not result in a significant, durable treatment effect in terms of weight-based symptom improvement in either adult or adolescent patients with AN, nor are they especially efficacious versus comparator treatments for psychological symptoms [30–31].

There are currently no FDA approved pharmacological treatments for AN, and medications are not considered a primary intervention given lack of clear efficacy [32]. However, the use of appropriate pharmacologic treatments such as antidepressants for conditions highly comorbid with AN, such as depression, anxiety, and obsessive compulsive disorder (OCD), is an important aspect of treatment [33–34]. Despite weak evidence for the use of psychotropic medications to promote weight gain, prevent relapse, and treat core features of AN, off-label prescribing of selective serotonin reuptake inhibitors (SSRIs) and/or antipsychotic medications for these purposes is common in clinical practice [35–37]. Neuromodulation may be considered in severe, refractory cases and appears to be safe in those with active comorbidities who would therefore otherwise be candidates for these treatments [38–39]. Electroconvulsive therapy (ECT) is sometimes considered for severe and enduring AN, including in adolescent patients, but evidence for this treatment, which can be invasive, expensive, and disruptive to the life of a youth, remains equivocal [40–42]. Transcranial magnetic stimulation (TMS) has been studied more extensively, though exclusively in adults, and with unclear benefit [43]. Several case reports/series on deep brain stimulation (DBS) in adults with severe AN have demonstrated markedly positive outcomes, but this treatment is highly invasive and difficult to access, there is no consensus on treatment protocols such as electrode placement, and long-term maintenance by a specialist is required [43].

Severe AN may, in some cases, warrant urgent involuntary treatment. For severely underweight patients who are hemodynamically unstable and refuse to engage in nutritional rehabilitation, this may involve involuntary nasogastric tube feeding, and in some instances the use of physical restraints, to achieve the rapid weight restoration necessary for medical stabilization. Enteral feeding over objection or via court order is a contentious topic with serious ethical implications in adults. This becomes even more complex in children and adolescents, as they typically lack the legal ability to consent to treatment in general, and a court order is not required for treatment without patient assent as long as parental consent is obtained. Some consider forced interventions to be ethically and clinically justified when a patient’s decision-making capacity is impaired, when the risk of death or serious morbidity is high, and when the likelihood of benefit outweighs the risk of harm. This is especially pertinent when considering that medical complications are sometimes reversible with early intervention in adolescents. Given the limitations of existing therapies, and the often refractory nature of the disease with high rates of chronicity and relapse, there is a distinct need, and even moral imperative, for new treatment approaches for AN in young people.

Interest in less invasive neuromodulatory and novel pharmacologic interventions has therefore grown. One such approach is the use of ketamine, a dissociative anesthetic with rapid-acting antidepressant properties. A review of the available literature identified 22 adult patients with EDs who received ketamine, either as a stand-alone treatment or in conjunction with psychotherapy [44]. Reported benefits were heterogeneous, but generally included improvements in comorbid depression, suicidality, anxiety, and obsessive thinking. Several reports also described partial, complete, and/or sustained remission in ED symptoms, such as restrictive eating, binge-purge frequency, and cognitive rigidity. In some cases, these changes were accompanied by weight restoration and resolved amenorrhea. A separate retrospective analysis of eight ED patients found significantly improved BMI following ketamine treatment, with a trend toward greater weight regain beginning after the fourth or fifth session [45]. Individual and group-based models of ketamine-assisted psychotherapy (KAP) have also been evaluated as strategies to maximize or prolong ketamine’s therapeutic effects. For instance, a recent case study reported that KAP, delivered within an Acceptance and Commitment Therapy (ACT) framework, was associated with rapid and sustained reductions in ED psychopathology at 5-month follow-up in a patient with AN [46]. Although the evidence remains limited, these findings suggest that ketamine may hold promise for treating EDs, particularly when combined with individual or group-based psychotherapy. This emerging clinical interest further reflects a broader shift toward investigating psychedelic and psychedelic-adjacent compounds (like ketamine) for EDs, as these treatments may offer a novel route to addressing the entrenched cognitive, emotional, and behavioral rigidity characteristic of AN as well as common psychiatric comorbidities.

## The Standard Psychedelic-Assisted Psychotherapy Model

Empirical support for psychedelic-assisted psychotherapy has developed in two major waves. The first, spanning the 1950s to early 1970s, was driven by academic psychiatrists who conducted thousands of administrations of LSD and psilocybin in clinical settings. Despite promising reports—especially for mood and substance use disorders—the research was undermined by methodological flaws and societal backlash, culminating in the 1970 Controlled Substances Act, which halted nearly all studies [47, 48]. The second wave began in the 1990s, with rigorous early-phase studies of psychedelics like N, N-dimethyltryptamine (DMT) and psilocybin in healthy volunteers that re-established safety and revived the therapeutic framework emphasizing preparation, supportive environments, and integration.

Since 2010, research has increasingly focused on clinical populations, with small trials showing substantial and lasting benefits for conditions such as depression, addiction, and cancer-related distress [48–53]. Meanwhile, MDMA (3,4-Methylenedioxymethamphetamine)-assisted therapy for post-traumatic stress disorder (PTSD), led by MAPS (now Lykos Therapeutics), advanced to late-phase trials and received Breakthrough Therapy designation from the FDA, though a 2024 new drug application was rejected due to data-integrity concerns. The field is now shaped by industry-backed, multi-site phase 2 and 3 trials aimed at regulatory approval, with ongoing studies targeting a broad range of mental health conditions and continued momentum despite regulatory hurdles.

The model that dominates contemporary clinical research with psychedelics has crystallized into a fairly uniform, three-stage arc: preparation, dosing, and integration [6, 54]. After rigorous medical and psychiatric screening, each participant is paired with at least one, but often two, trained clinicians—usually referred to as facilitators or monitors—who remain a constant presence throughout treatment. During this time the team gathers the patient’s biopsychosocial history, clarifies goals, and introduces core ideas such as *set* (the participant’s mindset, expectations, intentions, and psychological state) and *setting* (the physical, interpersonal, and cultural treatment environment). Set and setting are key concepts in psychedelic-assisted therapy, usually discussed early and often with patients, and used to frame preparation, guide therapeutic intention-setting, and intentionally structure the physical, interpersonal, and emotional environment in which dosing occurs. The aim is to maximize psychological safety, therapeutic alliance, and the likelihood of adaptive meaning-making during and after the psychedelic experience, optimizing outcomes. Rapport-building is paramount in the early phase of treatment, because the patient will soon enter an acutely vulnerable, altered state; thus the sessions also rehearse coping strategies, establish safety plans, and discuss the full range of possible emotional and perceptual experiences.

The dosing session itself is conducted in a room intentionally furnished to feel like a comfortable home-like environment. Patients recline on a couch or bed, don eyeshades, and listen to curated music. The two facilitators adopt a largely non-directive stance, offering verbal reassurance or gentle touch (with prior consent) when clinically indicated.

In the days that follow, integration visits give patients an opportunity to revisit the material that surfaced during dosing, explore its personal meaning, and translate insights into actionable change. These sessions—often totaling many therapist-hours in the acute treatment window—are widely considered indispensable to durable benefit, yet they complicate attempts to parse the drug’s specific contribution from that of psychotherapy [55]. Critics argue that drug and talk therapy are so entwined in this model that isolating pharmacological efficacy requires alternative designs or control conditions (often an inactive placebo, low-dose psychedelic dosing, or active placebos like diphenhydramine) but given the unique psychoactive effects of psychedelic substances effective blinding is notably difficult [56].

Some contemporary trials explicitly include a specific therapeutic modality in the prep/integration phases, such as cognitive behavioral therapy, motivational interviewing, or ACT [57]. The feasibility of the two-facilitator model has been questioned due to personnel burden and associated costs, and alternative models have been proposed, including individual facilitator and group therapy models, to enhance scalability and accessibility [58, 59]. These models will likely continue to evolve as the field balances safety and efficacy with broader accessibility.

## Psilocybin-Assisted Psychotherapy for Anorexia Nervosa

### Mechanisms of Action and Theoretical Basis

Research on the mechanisms of PAT spans multiple levels of explanation from molecular and neural substrates to cognitive, emotional, and experiential processes. This reflects the complexity of psychedelic effects and their potential transdiagnostic efficacy, and highlights several dimensions through which the potential risks and benefits of PAT may be evaluated in populations of interest. As is the case with many mental health conditions, the pathophysiology of AN is not fully elucidated, and as such our discussion of the potential mechanisms is speculative. Here we review some of the extant literature.

Psilocybin is a prodrug that undergoes rapid hepatic metabolism to psilocin, the primary active compound responsible for its psychoactive effects. At the level of neurotransmission, psilocybin's effects are thought to be mediated primarily via 5HT-2 A receptor agonism [60]. Data connecting dysfunctional serotonin (5HT) signaling to AN is equivocal, but some implicate altered functioning that could hypothetically be augmented by 5HT-modulating agents, especially considering 5HT'st role in anxiety, behavioral inhibition, body image distortion, appetite, and memory [61–63]. People with AN may have altered 5HT-2 A binding patterns, including diminished 5HT-2 A receptor functioning, particularly in the frontal cortex [64, 65]. AN may also be associated with differences in allele frequencies of a serotonin receptor promoter gene, though data are mixed [66].

Functional neuroimaging studies suggest that psilocybin may potentiate changes in resting state functional connectivity within the default mode network, possibly normalizing patterns within these systems [67]. At network levels, AN is characterized by increased resting state functional connectivity between the default mode network and frontoparietal network and angular gyrus, associated with disease persistence and interoceptive awareness, respectively [68]. Functional connectivity in the brains of people with AN tends to become increasingly modular as the disease progresses, and adolescents with AN have been found to have reduced connectivity within and between major cortical and subcortical resting state networks [67–72]. Therefore, psilocybin may intervene on the level of systemic neurological organization to normalize network connectivity, restoring more optimal processing.

A proposed unifying explanation for the observed transdiagnostic utility of classical psychedelic medicines, including psilocybin, is their unique anti-inflammatory mechanism [73]. Systemic inflammation is thought to be a risk factor for psychiatric illness, including EDs, and there is evidence that pro-inflammatory states contribute to appetite suppression and are therefore theorized to mediate the cycle of malnutrition, rigid cognitions, and feeding behaviors that characterize AN [74–76]. Given these effects, there is a theoretical basis for the possibility that 5HT-2 A receptor agonists like psilocybin effect change at a systems level by reducing inflammatory processes and improve outcomes in AN.

Preclinical evidence also suggests that some psychedelics, including psilocybin, may reopen critical periods of social reward learning, and PAT could help recalibrate dysfunctional reward systems by weakening core prior beliefs and restoring contextually-appropriate hedonic responses [77–78]. Maladaptive reward processing is thought to play a role in AN, characterized by normally rewarding food-related stimuli being processed as aversive; conversely, individuals with AN have demonstrated paradoxically increased activation in reward-related neural circuits in response to aversive stimuli, such as images of moldy food [79, 80].

Psilocybin use has been associated with enhanced cognitive flexibility, the capacity to shift thinking between mental sets, reduce perseveration, and adapt behavior in response to changing rules or feedback. This may be particularly relevant in the treatment of AN, a condition marked by extreme cognitive rigidity. The idea of improved cognitive flexibility incited by psilocybin is well-supported by preclinical models, and a mediating effect on eating behaviors has been demonstrated [81, 82]. For instance, female rats exposed to an activity-based anorexia paradigm, psilocybin improved reversal learning (helping animals adapt more readily when reward contingencies changed), and was associated with better weight maintenance under anorexia-like conditions [82]. Improvements in cognitive flexibility have likewise been demonstrated in humans by Doss and colleagues (2021), who found that in patients with MDD, psilocybin reduced perseverative errors on cognitive tasks for up to four weeks post-treatment (though this did not directly correlate with mood improvement) [83].

Psilocybin may also exert therapeutic effects by enhancing psychological flexibility, a core construct in ACT, defined as the capacity to remain in contact with present-moment experience and to persist or change behavior in the service of personally held values, even in the presence of difficult internal experiences [84–86]. Psychological flexibility is a transdiagnostic construct described as an essential set of processes that support adaptive functioning. In clinical trials, improvements in psychological flexibility after psilocybin use have been associated with reductions in a range of psychiatric symptoms including depression and anxiety [87–89]. Psychological flexibility is inversely correlated with disordered cognitions and behaviors around eating and body image, and lack of body image psychological flexibility has been associated with disordered eating behavior and found to predict relapse and quality of life following treatment [90, 91].

One explanation under the REBUS (RElaxed Beliefs Under pSychedelics) model posits that psychedelics temporarily “relax” the precision of high-level priors in the brain’s predictive coding hierarchy, allowing for increased bottom-up information flow and the revision of maladaptive beliefs [92, 93]. This relaxation of rigid priors may be particularly relevant for AN, where inflexible beliefs about food, weight, and control are often deeply entrenched and resistant to change. By increasing both neural and psychological plasticity, psilocybin may open a therapeutic window during which patients are more able to engage with and integrate new cognitive and emotional perspectives, in this case particularly around body image and overall self-evaluation, entrenched beliefs related to worth and purpose, and insight into the seriousness of their illness.

Further, psilocybin has been shown to occasion profound spiritual or mystical type experiences, characterized by feelings of unity, transcendence of time and space, ineffability, and a deeply felt sense of sacredness [94]. Such states have been associated with lasting positive changes in mood, values, and behavior [95–97]. The intensity of the mystical experience has been correlated with longer term behavioral and mood outcomes, suggesting that these experiences can serve as catalysts for enduring shifts in perspective, emotional processing, and behavior. In the context of AN, where rigid belief systems and a deeply entrenched sense of identity are central to the illness, such experiences may offer a renewed sense of meaning and self that supports recovery.

Taken together, these mechanisms, including agonism at the 5HT-2 A receptor, modulation of large scale brain networks, anti-inflammatory effects, and promotion of neural and psychological plasticity, may uniquely position psilocybin as a promising candidate intervention in individuals with AN. When provided in a supported context, these effects may converge to enhance cognitive flexibility, disrupt entrenched maladaptive beliefs, and facilitate meaningful shifts in identity, self-concept, and behavior that are difficult to achieve with existing therapies. While these mechanisms provide a compelling rationale, clinical evidence remains in its early stages, particularly for younger patients.

### Review of PAT for AN

High quality studies of PAT for AN and other EDs remain limited, and the literature has largely consisted of studies and case reports of patients aged 18 years or older. Early literature includes a 1959 case report of a 35 year-old woman with AN, likely binge/purge subtype (BMI 14 kg/m^2^), who received two doses of psilocybin for both mood and eating symptoms with “definitive recovery.” Changes in her ED symptoms were not reported in detail though she is described as putting on 7 kg of weight following treatment [98]. More recent qualitative studies have examined psilocybin and other classical psychedelics, like ayahuasca and LSD, for EDs [99–101]. Survey data also suggest that naturalistic psychedelic use is associated with reductions in disordered eating and improved mood and wellbeing in adults with a self-reported lifetime history of an ED [102, 103]. For instance, LaFrance and colleagues (2021) found an association between frequency of psychedelic use and reductions in disordered eating, mediated by spirituality and emotional regulation [103].

Recently, Ragnhildstveit et al. (unpublished) reported on a 23-year-old woman with severe, treatment-resistant AN, restricting type, who underwent psilocybin-assisted emotion-focused therapy (PAT-EFT) over a 12-week period. Structured within a parallel care model, the intervention comprised two high-dose psilocybin sessions (25 mg each) administered at a state-regulated psilocybin service center, alongside emotion-focused therapy (EFT) delivered in private practice outside the center. In total, the patient received five preparation sessions (three EFT-based, two facilitator-led), two psilocybin dosing sessions (non-directive, facilitator-led), eight integration sessions (six EFT-based, two facilitator-led), and one closing session (EFT-based). Psilocybin was well-tolerated with no serious adverse events reported. Scoring on the Eating Disorder Examination Questionnaire (EDE-Q) showed statistically reliable improvements in ED psychopathology from baseline to 1-month post-treatment (RCI = 3.90), which were maintained at 4-months follow-up (RCI = 5.62) [104]. These preliminary findings suggest that psilocybin combined with EFT may promote meaningful reductions in ED symptoms among young adults with longstanding, treatment-refractory AN, although there is a need for replication in larger and controlled trials.

There have also been several clinical trials investigating the use of PAT for AN in adults and young adults. The Center for Psychedelic Research at Imperial College completed a clinical trial including 21 female participants with AN who received three doses of psilocybin (10 mg and 25 mg) over a 6-week period delivered in a therapeutic environment which included preparation and integration sessions with trained therapists [105]. Preliminary published findings suggest that the treatment was well tolerated and led to statistically significant reductions on ED psychopathology as measured by global scores on the Eating Disorder Examination (EDE) in 14 participants, which was demonstrated by a preliminary ANOVA (*p* < 0.001, d = 1.134). Findings from the 12-month follow-up period have not yet been reported, however these results suggest that psilocybin treatment with multiple doses is safe and may be effective for those with active AN [105, 106].

Knatz and colleagues (2023) conducted a Phase 1 open–label pilot study evaluating safety and efficacy of a single 25 mg dose of synthetic psilocybin administered with psychological support (preparation and integration sessions, and support during the dosing experience) in 10 participants who met criteria for AN [107]. This study included participants in partial remission with continued symptomatology and those with active AN. There were no serious adverse events reported in the study sample. 40% of participants achieved cognitive remission from ED-specific psychopathology and significant reductions in shape and weight concerns were found, which persisted at 3-month follow-up. Notably, no significant effects were reported in weight restoration for underweight participants. Despite the small sample size and the uncontrolled design, outcomes suggest a therapeutic dose of psilocybin supported by trained therapists is safe, tolerable, highly acceptable, and potentially efficacious in reducing ED pathology in a subset of participants with AN [107, 108].

Gukasyan et al. (in review) completed a Phase 1 study of up to four doses of psilocybin (20–30 mg), combined with a psychological support based in motivational interviewing, in participants with at least a three-year history of AN and at least one prior treatment attempt. Among 19 participants, preliminary findings demonstrated medium to large effect sizes for improvements in quality of life, ED symptoms, and mood at one-month follow-up [109]. Subjective effects were generally rated as less intense and meaningful than in prior studies of psilocybin in other populations, aligning with anecdotal reports of reduced subjective drug intensity in individuals with EDs. Mild adverse effects such as nausea and headache were common, with few cases of transient elevations in blood pressure or heart rate. While serious medical events were anticipated due to the medically complex nature of the population, two noteworthy incidents occurred: one case of symptomatic bradycardia requiring hospitalization a week post-treatment, and one case of syncope during a dosing session. Despite these events, the intervention was generally well-tolerated and found to be acceptable by participants [109].

UCSF is also currently running a clinical trial using psilocybin with psychotherapy for young adults (aged 18–25) with AN (NCT06399263) that incorporates caregiver involvement. The study aims to enroll participants with various levels of acuity, including all genders, and Spanish-speaking families. Participants with a BMI as low as 12 are eligible, pending they demonstrate medical stability including on serum laboratory assessment, vital sign evaluation, physical exam and electrocardiogram, among other metrics.

### Theoretical Rationale, Developmental Context, and Safety Considerations for PAT in Adolescents with AN

There is an urgent need to identify alternative treatment modalities given the enormous burden of undertreated adolescent AN, and PAT may be one such model given relative safety and tolerability found in clinical trials of adult populations to date. However, understanding how the diverse mechanisms of psilocybin may operate in adolescents with AN requires careful consideration of developmental context.

#### Early Intervention and Theoretical Rationale

Although the majority of deaths due to AN occur later in the disease course [27], onset is frequently in adolescence, and those who develop this disease earlier in life have the poorest outcomes, including higher rates of chronicity and mortality [110, 111]. Progression of AN can result in serious medical complications and functional decline, and it is known that treatment closer to symptom onset is most likely to be effective [30]. Therefore, it is crucial to intervene effectively when an adolescent presents with AN to prevent progression, chronicity, development of comorbidity, social, academic, emotional, and developmental challenges, and in too many cases, death. PAT used in adolescent populations, if safe and effective, could be one way to intervene before AN progresses, becomes chronic, and/or results in more serious sequelae.

There is mounting evidence for the use of PAT for the treatment of disorders that are commonly comorbid with AN like depression, substance use disorders, and OCD [4, 35, 112–114]. Therefore, there is reason to believe these co-occurring psychiatric symptoms could be alleviated to some degree by PAT in addition to treating core features of AN in this population early on, improving the quality of their lives and perhaps, by extension, also prognosis [110].

#### Safety and Clinical Risk Considerations for PAT in Adolescents with AN

Due to the novelty of this treatment modality and current legal status, there is no publicly available data regarding safety in clinical settings, and a paucity of information about outcomes after naturalistic use in adolescents. However, we can extrapolate to some extent from safety data from literature reporting on adult use, including in AN, and consider the unique characteristics of both a younger person’s biology and the context of the developmental stage.

Although even data reporting on naturalistic use outcomes is limited, results from one recent prospective survey study of 435 adolescents and young adults found significant improvements in psychological wellbeing after experience with a classical psychedelic (inclusive of psilocybin), but compared to adults results indicated they had more challenging experiences while dosing, such as significant anxiety or other psychological discomfort, and a higher reported incidence of persistent perceptual disturbances [115]. Other available survey data demonstrate mixed results regarding an association between lifetime use of classical psychedelics and suicidality, as psilocybin was associated with lower, and LSD with higher lifetime suicidal thinking, planning, and attempts [116].

Contemporary clinical studies of PAT have recruited primarily adults, and in many cases have limited recruitment to participants age 21 or older. Adolescents were included in research in the first wave, though poorly structured trials lead to a relative paucity of data in this population. The most substantial research in adolescent and pediatric patients was in what would now most likely be described as autism with children as young as five years of age receiving LSD [117]. Adverse events were poorly documented, and improvement in symptoms were vaguely reported. On the other hand, studies of children and adolescents in the União do Vegetal (UDV) church who partake in regular consumption of ayahuasca, an admixture containing the powerful classic psychedelic *N*,* N*-DMT, seem to have no significant neuropsychological differences and may have less likelihood of consuming alcohol compared to age matched controls [118, 119]. Notably, adolescents in this community most often take the sacrament with their parents and other community members.

At this time there are no planned trials of psilocybin in adolescents. However, when given in the setting of carefully controlled clinical trials, psilocybin has demonstrated a favorable safety profile even in medically ill adult populations such as those with terminal cancer [51, 53, 120]. Common acute side effects, when they do occur, are generally mild and transient, including headaches, nausea, vomiting, anxiety, and modest increases in blood pressure and heart rate [121, 122]. In general, the abuse liability of psilocybin is remarkably low, setting it apart from many federally scheduled substances [123]. However, as with any biological psychiatric treatment considered for application in younger populations, differences between adult and developing neurobiology should be taken into account.

It is also important to account for the physiologic consequences of malnutrition in patients with AN when considering PAT. Prior studies in participants with AN have required that participants be medically stable enough to be appropriate for outpatient care, specifically, that their medical status would not warrant inpatient admission for medical stabilization [107, 109, 124]. Initial screening should include adequate testing to rule out dangerous electrolyte abnormalities, cardiovascular instability, and clinically significant hepatic or renal dysfunction. Given that medical stability may be precarious in some patients, it is also vital to monitor participants on an ongoing basis for changes in medical status. This may include periodic measurement of weight, vital signs, electrocardiography, and electrolytes, with referral to higher level of care as indicated.

The most serious psychiatric risks of psilocybin include precipitation of psychosis and/or mania. Since adolescents are less likely than adult patients to present with fully declared symptomatology of serious mental illness, extensive family history and nuanced personal history of psychiatric symptoms suggestive of higher risk—such those characterizing prodromal or full psychosis, or observed period/s of manic or hypomanic symptoms—should be obtained. Also, while potentially transformative, participants and treatment teams should be aware of the potential impact of a profound shift in consciousness, such as mystical-type experiences, significant perceptual, emotional, and cognitive changes, and ego-dissolution often occasioned by psilocybin that may uniquely impact younger people. Hallucinogen persisting perception disorder (HPPD) is a rare but potentially debilitating condition characterized by persistent visual disturbances following psychedelic use. Given the diagnostic ambiguity and limited epidemiological data, it is also important to monitor for HPPD symptoms prospectively in clinical trials involving psilocybin, particularly in younger or vulnerable populations.

To minimize these risk, potential participants require psychiatric screening by a qualified provider and ongoing monitoring for new mental health symptoms, exacerbations in existing mental health conditions such as depression and anxiety, and careful ongoing assessment of suicidal ideation.

#### Developmental Considerations

Adolescence is a critical period of development marked by heightened neuroplasticity, ongoing identity formation, and increased vulnerability to both psychopathology and environmental influence. These features may present a double-edged sword in the context of PAT: on one hand, increased neural malleability could render adolescents more responsive to interventions aimed at promoting cognitive flexibility, reconfiguring maladaptive belief systems, and restoring hedonic responsiveness. On the other hand, the instability of self-concept and ongoing brain maturation raise concerns about the potential risks of introducing powerful, potentially destabilizing experiences, such as those occasioned by psilocybin, into a still-developing psyche.

In particular, the cognitive rigidity and entrenched belief systems characteristic of AN may be less fixed in adolescents than in adults, which could allow for greater responsiveness to interventions aimed at disrupting maladaptive priors and enhancing behavioral flexibility. However, this same developmental plasticity also necessitates a careful, scaffolded therapeutic approach that includes age-appropriate integration and robust support. Spiritual or mystical-type experiences, while potentially transformative, must also be considered in light of adolescents’ evolving capacity to interpret and integrate such experiences meaningfully. The therapeutic container, both in terms of interpersonal support and developmental appropriateness, must be strong enough to hold the psychological material that may be stirred.

### Suggested Adaptations to PAT for Adolescents with AN and Emerging Models

We propose several augmentations to how PAT is delivered to more effectively and safely serve the adolescent AN population, and suggest systematic testing of treatment protocols that include these adaptations to assess their feasibility and efficacy.

#### Family Involvement

Family involvement is the standard of care in adolescent treatment for AN, but is not necessarily part of PAT as it is currently being practiced. One often overlooked aspect of clinical trials is the profound impact of the environment to which a patient returns in the days or weeks after a psychedelic experience. Given that adolescents are still embedded in their family systems for emotional, logistical, and financial support, recovery often requires not only individual change but also a meaningful reorganization of family dynamics and structured support in the home environment. This opportunity for the family dynamics to shift may in fact be some of the most operative changes in the treatment of AN in this age group [125]. Without corresponding shifts in the relational field, therapeutic gains resulting from PAT may be compromised by returning the adolescent to the very context in which symptoms emerged or were reinforced. In the absence of caregiver support—such as validation, emotional containment, and openness to the adolescent’s evolving perspectives—the transformational potential of PAT may be blunted or, in some cases, even destabilizing both for the adolescent and for the family system [126]. In an ongoing clinical trial of psilocybin therapy for young adults with AN (NCT06399263), caregiver participation in both preparation and integration sessions is required, drawing on principles from Emotion-Focused Family Therapy (EFFT). While no single therapeutic modality has been established as superior in this context, this model offers one promising approach for structuring family involvement in PAT. EFFT emphasizes the caregiver’s role in supporting emotional processing and teaches specific skills—such as validation and mirroring—to help interrupt maladaptive patterns and reinforce emotional and cognitive flexibility [127]. Within this model, caregivers are prepared to understand the potential impacts of PAT, equipped with scripts to support emotion processing, and guided in practicing emotion coaching to facilitate further integration and processing in the home environment. This structured involvement aims to maximize the therapeutic potential of PAT by fostering a supportive family environment that reinforces insight, emotional openness, and behavior change following the psychedelic experience. Thus, engaging families not only mitigates potential risks but also provides a scaffolding for durable therapeutic change, especially as adolescents attempt to reintegrate novel ways of thinking and relating into their everyday lives. Situations involving current or unresolved abuse within the caregiver–adolescent relationship may represent a contraindication to this treatment approach [126].

#### Expanded Therapy Model

Generally, PAT models include several 90-minute preparation sessions and at least one to three integration sessions per dosing session [128]. However, for adolescents, we recommend additional preparation and integration visits. Compared with adults who are considering or starting PAT work, in addition to family work, younger people could benefit from additional skill-building, especially around recognizing and working with challenging thoughts and feelings, and emotional regulation. This may be done by borrowing from elements of dialectical behavioral therapy (DBT),ACT, cognitive behavioral therapy (CBT), and motivational interviewing (MI), tailored to the participant’s abilities, developmental level, and comorbid psychiatric concerns [129]. Likewise, a longer period of integration may be indicated for younger participants, especially those who present with more severe pathology related to their ED or other mental health or safety concerns.A multidisciplinary team, including psychotherapists, medical providers, and nutritionists, can help provide comprehensive support to scaffold the profound emotional, cognitive, and relational changes that psychedelic experiences may catalyze while ensuring that nutritional stability and safety are closely attended to.

Something that is conspicuously missing from many clinical trials involving PAT is ensuring appropriate follow-up for patients, especially considering the high risk of ongoing symptom burden and relapse into severe disease. One proposed augmentation to a typical PAT model, which typically concludes with return to community treatment after the last integration session, is to actively involve the patient’s previously and/or newly established care team throughout the intervention period. This approach may enhance continuity of care and support sustained therapeutic gains.

#### Enhanced Consent Process

In designing the consent process for adolescent PAT trials and implementing these treatments, it will be necessary to respect the importance of parental consent while also allowing for adolescent informed assent. For vulnerable individuals, including adolescents, an enhanced consent/assent process that goes beyond standard informed consent/assent used for initiating psychiatric medication or intervention, or is even typically carried out for adult PAT participants, is recommended.

While it is generally accepted medical practice to treat minors involuntarily in cases of severe, life-threatening complications of AN, it is essential that psychedelic therapies remain fully, unequivocally voluntary for all potential participants. Aside from the clear, extreme ethical concerns of coercive psychedelic treatment, the effectiveness of psychedelic therapy also depends heavily on participant readiness, willingness, and engagement. Administering psychedelics without full informed consent/assent is likely to increase the risk of adverse outcomes, such as heightened anxiety or other forms of psychological distress, some of which may extend beyond the period of acute drug effects [130]. This is liable to undermine the therapeutic potential of the intervention, and perhaps future treatment attempts in an often already ambivalent patient population. A number of ethical, medical, and developmental considerations must be accounted for to ensure participation is both informed and freely chosen [131, 132].

The ability to obtain true informed assent from an adolescent with AN for this type of treatment presents some unique challenges. First, adolescence is a transitional developmental period wherein patients are undergoing maturation in emotional and cognitive capabilities. As such, adolescents are ideally expected to assume increasing responsibility for their psychiatric treatment as they near adulthood. However, their decision-making and problem-solving capacities often differ substantially compared with adults, and some participants may exhibit additional delays in developmental maturity.

These challenges may be further compounded by cognitive deficits caused by AN itself. Specifically, general hypofrontality with related specific pathological neurocognitive patterns, including cognitive inflexibility, weak central coherence, and perseveration, may impair the ability to understand and synthesize information and make an informed choice [133, 134]. Given the cognitive effects of malnutrition, in addition to developmental considerations related to age and maturity, it is critical that information about PAT for AN is communicated clearly, accessibly, and comprehensively. It should be made explicitly clear to adolescent participants and their families the realities of the novelty of this treatment modality, at times ineffable and unpredictable effects of psilocybin, and possible short and long-term outcomes of the treatment. These elements of the treatment should be discussed at great length, with frequent assessments for understanding.

However, even if information is unambiguously conveyed, assessing this patient population’s capacity for informed decision-making may be challenging. As such, we recommend evaluating for capacity to assent at baseline and throughout treatment, and obtaining assent from the potential participant on an ongoing basis during the intervention, accounting for their active psychological developmental and often fluctuating medical and mental status. It is, though, equally important not to presume that adolescents with AN lack the capacity to provide informed assent to PAT on the basis of their age and/or ED. Just as patient consent and assent are essential to the extent possible based on legal, ethical, and medical principles, demonstrating commitment to an adolescent’s autonomy should be honored by taking into consideration their preference for type of treatment, especially when they are indeed judged to be a capable decision-maker [135].

Current and/or historical approaches to an adolescent’s ED treatment may have involved less than sovereign or even compulsory, like caregiver- or treatment team-led nutritional restoration, and in some cases, involuntary feeding. Adolescents may also experience more subtle forms of provider- or caregiver-sanctioned coercion, such as conditional access to education or extracurricular activities, or other valued opportunities contingent on treatment participation. Therefore, efforts must be made to ensure that consent and assent are free from undue influence or coercive contingencies, however well-intentioned they may be, taking into account the sometimes complex dynamics of choice and freedom that have developed in the context of treatment for AN in young people. Independent interviews with the adolescent are therefore essential to ensure that assent is being freely given. Furthermore, motivation for treatment engagement should be assessed prospectively with clear protocols established in the event a participant withdraws assent to help to uphold ethical standards and protect the autonomy of adolescent participants.

This more robust and developmentally sensitive process of obtaining informed consent from the family and assent from the adolescent (who is deemed to have capacity to make this choice) is necessary to ensure true, informed, and ongoing permission is granted by all to move forward with the PAT treatment process.

### Other Practical and Ethical Considerations

#### Justice and Access

AN was once conceptualized as a disease of young, white, middle- and upper-class biological females. However, data now suggest that boys, youth of color and with indigenous heritage, and sexual and gender minority youth have been systematically underrepresented in diagnostic, treatment, and educational efforts [11, 136]. Similarly, treatments involving psilocybin and associated therapies have also largely failed to recruit demographically diverse study teams and participants [137]. Further, the “psychedelic renaissance,” including the academic, social, and political movement toward wide adoption of these therapies, is haunted by the historical context of the war on drugs and disproportionate criminalization of marginalized populations under the guise of public safety as well as cultural appropriation of plant-based psychedelics from minority communities [138]. It will be essential that clinical trials of PAT, should they expand to include younger patients, prioritize the inclusion of diverse populations and address the needs of historically excluded communities. This will require culturally responsive needs assessments, multilingual providers, community involvement, and active and ongoing commitment from clinicians, investigators, and policymakers alike.

#### Patient Selection

A thorough screening process will be crucial to identify appropriate candidates for PAT in adolescents with AN. Ideal candidates are likely to possess some degree of psychological insight into their illness and motivation to utilize the psychedelic experience, therapy, family involvement and follow-up care towards their recovery. Adolescents with more advanced psychological development such as the capacity for abstract reasoning and emerging metacognitive skills may be better equipped to engage meaningfully with the altered states of consciousness occasioned by psychedelics. Early adolescents may be less appropriate until further data emerge. There could be a role for this type of treatment in individuals who are less medically and/or psychologically stable given the dangerousness of illness progression, including high mortality rates. However, given the potential psychological intensity of PAT, and the need to remain actively engaged in a potentially lengthy treatment course, its use in patients with precarious medical stability, acute suicidality, or impaired reality testing should be approached with considerable caution, especially in adolescents or young adults. Further research will be needed to evaluate the safety and efficacy of PAT in adolescents across varying levels of illness severity. It is possible that patients who are somewhat weight- restored may be in a better psychological and neurochemical state to make use of PAT, though this has yet to be empirically demonstrated.

#### Patient and Family Inclusion in Research and Treatment Models

Often, individuals and families with lived experience are left out of important discussions around research and implementation. As clinical trials and treatment protocols are designed and carried out, it will be important to include their voices, eliciting preferences, ideas, and concerns. For instance, some studies have used patient and family advisory boards, and we encourage such practices. Existing surveys and qualitative research also offer valuable insights that can inform study design, and we support ongoing systematic collection of this type of information [139]. Ultimately, the goal of empirical study is to inform the development of real-world treatment to help individuals, families, and communities heal from devastating illness, and it is imperative we work together toward safe, inclusive, accessible models of care that align with the true need.

## Conclusion

AN in is a debilitating and often life-threatening illness with typical onset in adolescence. A substantial portion of patients fail to achieve durable response to existing therapies. The emerging promise of PAT in related psychiatric conditions has prompted interest in its potential for treating AN. However, applying this approach to adolescents demands a cautious and methodical path forward. Adolescents differ meaningfully from adults in their developmental stage, medical vulnerability, and legal status, necessitating tailored protocols and stringent safeguards. Substantial empirical work is still needed to clarify for whom, under what conditions, and in what formats PAT may be both safe and effective. Priorities for future research include examining family-based models of care, optimizing psychotherapeutic frameworks, assessing safety in medically and psychiatrically complex cases, and ensuring diverse and inclusive participant samples.

Any future implementation must uphold the highest ethical standards, including robust screening and monitoring, enhanced consent and assent procedures, and developmentally appropriate support. Researchers, clinicians, and families must work collaboratively to ensure that any exploration of this intervention remains grounded in evidence, caution, and a commitment to the well-being of young people.

## Key References

- Madden K, Flood B, Young Shing D, et al. Psilocybin for clinical indications: A scoping review. Journal of Psychopharmacology. 2024;38(10):839-845. 10.1177/02698811241269751.
  - ○ This article emphasizes the potential of psilocybin in treating a variety of psychiatric and other disorders, highlighting the need for additional research in this area.
- Swieczkowski D, Kwaśny A, Pruc M, Gaca Z, Szarpak L, Cubała WJ. Efficacy and safety of psilocybin in the treatment of Major Depressive Disorder (MDD): A dose-response network meta-analysis of randomized placebo-controlled clinical trials. Psychiatry Res. 2025;344:116337.
  - ○ This article establishes the safety and efficacy of the use of psilocybin in the treatment of depression and potential for efficacy in treating other disorders.
- Koning E, Brietzke E. Psilocybin-assisted psychotherapy as a potential treatment for eating disorders: a narrative review of preliminary evidence. Trends Psychiatry Psychother. 2024;46:e20220597. 10.47626/2237-6089-2022-0597.
  - ○ This article suggests potential efficacy of PAP in treating AN.
- Downey AE, Chaphekar AV, Woolley J, Raymond-Flesch M. Psilocybin therapy and anorexia nervosa: a narrative review of safety considerations for researchers and clinicians. J Eat Disord. 2024;12(1):49. 10.1186/s40337-024-01005-z.
  - ○ This article establishes preliminary safety data for the use of psilocybin and PAP in treating anorexia nervosa
- Lacroix E, Fatur K, Hay P, et al. Psychedelics and the treatment of eating disorders: considerations for future research and practice. J Eat Disord 12, 165 (2024). 10.1186/s40337-024-01125-6.
  - ○ This article details ethical considerations in the study of psilocybin and psychedelics and presents provisional guidelines for research in this area of increasing interest.

## Data Availability

No datasets were generated or analysed during the current study.
